# How Medical Students Negotiate Their Roles in the Figured World of Curriculum Co-Creation: A Longitudinal Qualitative Study

**DOI:** 10.5334/pme.2538

**Published:** 2026-03-25

**Authors:** Emmanuel Tan, Jennifer Cleland, Jessica Ang, Russell Yap, Siew Ping Han

**Affiliations:** 1 Lee Kong Chian School of Medicine, Nanyang Technological University, Singapore; 2 Nanyang Technological University, Singapore

## Abstract

**Introduction:**

Co-creation, where students and faculty partner to develop curricular materials and learning, is increasingly recognised for its potential to enhance student engagement, ownership, and inclusivity. However, little is known about how students experience and navigate co-creation partnerships. Understanding this process is essential to maximise understanding of, and gains from, student-faculty partnerships. To address this gap, we explored how medical students navigate their roles and boundaries within the process of co-creation.

**Methods:**

This is a longitudinal, qualitative study, informed by a constructivist worldview. We conducted 15 semi-structured interviews with five students across three timepoints and collected supplementary data from students’ reflective writing. Initial data analysis was thematic and iterative. We then used Figured Worlds theory to explore how co-creation enabled medical students to author identities, negotiate positionalities, and engage in world-making.

**Results:**

We identified three main themes. Students wanted to contribute but had to negotiate legitimacy with faculty within the co-creation figured world. They authored new identities as curriculum stakeholders, moving beyond learner advocacy to engage with broader curricular perspectives. They had to navigate tensions at the boundaries between being learners, collaborators, and future doctors, whilst negotiating constraints imposed by institutional structures and power relations.

**Discussion:**

Students authored identities as advocates, collaborators and aspirational future doctors, and moved across multiple figured worlds during the co-creation process, negotiating identities and the tensions that emerged through this process.

## Introduction

Collaborative approaches to the design and delivery of teaching and learning have gained increasing attention within health professions education in recent years [1,2,3]. One such approach is co-creation, defined as a collaborative process where students and staff work together to create or develop curricula or instructional materials (such as instructional content or study guides [4]). In co-creation, students bring valuable perspectives to ensure materials resonate with learner needs and experiences. This process of student-faculty collaboration has been shown to deepen student engagement, foster a sense of ownership, and cultivate a more inclusive learning environment [3,5].

However, despite the growing recognition of students’ potential contributions to co-create curricula and educational materials, we know surprisingly little about how students navigate their roles, boundaries and the inherent power dynamics involved in the co-creation process. This is particularly pertinent in the highly structured and regulated milieu of medical education [6], where students are “trained” and “assessed” by faculty and clinicians and where hierarchical structures can lead students to view faculty as authority figures rather than as collaborators, potentially hindering co-creation efforts [7]. In other words, while co-creation invites students to contribute as collaborators, their legitimacy in this role and context may be constrained by wider societal perceptions of expertise and structural/systems norms [8,9]. Additionally, while it seems logical to assume that relationships within the co-creation process evolve over time, there is a paucity of longitudinal research on this topic (longitudinal studies on co-creation have largely focused on co-creation with patients rather than students [10] or have been carried out in other contexts [11,12]).

Given this, it is critical to examine how students enter and navigate the process of co-creation over time, and in so doing, explore how co-creation is experienced by participating students. Understanding this is essential to maximise understanding of, and gains from, student-faculty partnerships, and to inform the design and implementation of co-creation initiatives.

To address this gap, we explored how medical students navigate their roles and boundaries within the process of co-creation. Our specific research question was: How do medical students negotiate their roles, boundaries, and tensions within the figured world of co-creation?

## Methods

We drew on Figured Worlds [13] as a theoretical framework to examine the experiences of medical students in co-creating educational materials. Broadly, Figured Worlds describes social contexts where one’s actions and behaviours are shaped by cultural norms, expectations and power relations [14,15]. Students entering the Figured World of co-creation need to navigate complex role expectations that may not align with their traditional positioning as learners. These tensions parallel those identified by Bennett *et al*. [16] where students needed to navigate between different “figured worlds” in authoring their own identities of what it means to be a good doctor. Figured World theory thus offers insight for students entering the figured world of co-creation, where one world ascribes to the traditional role expectations of students as (relatively passive) learners while the other promises the collaborative ideals of co-creation where students are active contributors to medical education.

### Study Design

This study adopts a qualitative research design underpinned by a constructivist worldview [17]. By emphasizing the social and cultural construction of meaning, social constructivism can shed light on how people learn to interpret and navigate the different figured worlds they inhabit.

We used two data sources to explore how students navigated their roles, boundaries, and power dynamics in the co-creation of educational materials. Our main source of data was semi-structured, longitudinal interviews with students involved in co-creation initiatives to gain insight into how they negotiated their roles. The longitudinal design was adopted to examine how identity construction and role negotiation unfolded over time [18,19]. Our second source of data was reflective narratives about the co-creation experience written by the same students.

### Context and recruitment

The study was conducted at the Lee Kong Chian School of Medicine (LKCMedicine), Nanyang Technological University (NTU), Singapore. LKCMedicine offers a five-year undergraduate medical programme, which follows an integrated, systems-based curriculum.

The co-creation initiative was conducted alongside the scholarly project, a mandatory six-week research component of the fourth-year curriculum. Students are offered a choice of scholarly projects from a list offered by faculty. Students rank projects according to preference and are assigned a project accordingly. Across 2022 and 2023, we offered five co-creation projects, all of which were taken up by students. In three projects, students worked with faculty to gather data to refine the content and delivery of an endocrine module (2022). The other two students were engaged in a project aimed at enhancing science practical sessions (2023). While the scholarly project itself was mandatory, it was made clear to the students that taking part in research about co-creation was separate to the scholarly project itself, completely voluntary and would have no impact on their project progression or assessment.

### Data collection and analysis

Fifteen semi-structured interviews were conducted, three with each participating student. Each participant was interviewed at the start and end of their six-week co-creation scholarly project, and about six months after project completion. The first two interviews were intended to elicit initial perceptions, and the third to capture students’ retrospective reflections on how the co-creation experience may have influenced their sense of professional identity and role(s) over time (see Supplementary Appendix 1). This longitudinal dimension is consistent with the study’s use of Figured Worlds to examine identity negotiation over time.

The written reflections were gathered once, at the end of the six-week scholarly project, to gain deeper insights on the participants’ experiences of co-creation. These entries were collected for research purposes (they were not part of the scholarly project requirements). Students were provided an initial briefing and a guiding question to structure their reflections (see Supplementary Appendix 2). These were developed from the dimensions of the Agency of University Students Scale [20], which includes questions on the agency, participatory and collaborative aspects central to co-creation experiences. These dimensions aligned with our interest in understanding how students may navigate roles and identities within the figured world of co-creation. No length requirement was imposed.

Each data source served a distinct analytic purpose within the Figured Worlds framework. Longitudinal interviews traced identity development over time, while the reflective writing provided insight into their sense of agency and identity.

Initial data analysis was inductive and thematic [21]. The analysis followed an iterative and collaborative process driven by the aim to achieve interpretive depth. RY first reviewed the data and identified an initial set of themes, which was further refined through multiple team meetings with ET and HSP to refine interpretations and resolve any discrepancies. ET and HSP then conducted a secondary analysis adapting Bennett *et al*.’s [16] analytical approach, which examined how students positioned themselves across figured worlds, exercised agency, and authored new identities. The data sources were each reviewed, with themes compared and integrated across sources in the collaborative team analysis.

### Reflexivity

Our approach to the study and data analysis was shaped by our differing positions in relation to the medical school and the data. ET, HSP, JA and JC were medical school faculty. RY was a student researcher but from another department and so had no knowledge or experience of medicine/medical education. HSP directly supervised the scholarly project students. JA, who was not directly involved in supervising the scholarly projects, conducted all the interviews. We all valued the idea of co-creation but also held slightly different perspectives on what this could look like, and the relative role of faculty and students in co-creation. We also had different positions in terms of how and where we ourselves were educated (e.g., Singapore, UK) and our own experiences of engaging in anything which could be considered similar to co-creation as learners. The team’s varied positions were discussed to sustain a reflexive stance, and these diverse and differing positionalities supported and enriched our analysis of the data.

### Ethical approval

The study protocol (IRB-2021-1070) was approved by the Institutional Review Board of Nanyang Technological University, Singapore.

## Results

We identified three main themes that illustrate how students navigated and authored identities across different figured worlds during the co-creation process. These are discussed below with the use of quotes to aid confirmability.

### Negotiating legitimacy within the co-creation figured world

The participants were surprised when faculty listened to and valued their input about their proposed changes to the educational materials and curricula. They initially struggled to view themselves as legitimate actors within the figured world of co-creation, where the roles, rules, and social relationships governing curriculum development were largely unfamiliar to them.

“*I was very pleased and surprised to see how much they were actively engaged in our discussion (and) what we brought to the table, which made me feel a lot more confident about what we are doing*.” (P03, Second Interview)

This surprise was also seen in the reflective diaries. Students documented their shifting sense of who they were within this new figured world, the different norms and expectations from those of the conventional student role. That their suggestions were taken seriously and even incorporated into the final outcomes challenged their initial assumptions about the boundaries between faculty and student roles, and the figured worlds each inhabited. For example, one student wrote, “*I felt like I was treated like an equal. I was very glad that the content experts were very receptive to what we had to share and even engaged in deep discussion with us on the feasibility of the various suggestions on how to improve*.” (P02, Reflection Journal)

Students described a growing sense of ownership as they realised that their contributions had a tangible impact on shaping the project’s direction. They began to view themselves not only as contributors but also as stakeholders in the project’s progress. As they began to see themselves as legitimate participants, rather than peripheral observers, they started to reposition themselves as advocates for their peers (see below).

### Authoring new identities across figured worlds

Participants initially positioned themselves as engaging in co-creation to improve the curriculum, help future cohorts and give something back to the School. This stemmed from their thinking that the programme’s pre-clinical content did not fully align with, or reflect, the challenges they believed students encountered during their clinical rotations: “*I think this project, as well as the co-creation process, is very interesting as it provides a way for students to now help in shaping the curriculum for future batches. I think that aspect of both being able to show our gratitude to the school curriculum, as well as potentially helping students have their voices heard, motivated me to give my best in this project*.” (P01, Reflection Journal)

This advocacy role was continuously negotiated throughout the clinician-student discussions about learning materials. Students questioned how much theory is truly necessary, who it is meant for, and the rationale behind the current structure of the curriculum. The process of co-creation drew students into the figured world of curriculum design and delivery, and entered a world with its own artefacts, norms, and social relationships. We also sensed a subtle shift in how students authored their identities when they crossed into this figured world. Specifically, they moved from a predominantly student-centred view toward a more complex identity as legitimate actors who could interrogate and contribute to the curriculum.

“*Because I was …. asking the doctor like why did we put that in the curriculum and why not this. It makes me look back and think why am I being taught this in this way … this (co-creation) project made me realise everything in the curriculum is there for a reason, and [gave me a] better appreciation and understanding on the process behind the curriculum*.” (P01, Third Interview)

### Navigating figured world boundaries: Identity tensions and ongoing negotiation

However, while students expressed excitement and felt empowered at being able to contribute meaningfully to the project, we also found moments of hesitation, doubt, and self-questioning. There were tensions between embracing their emerging agency and role in co-creation and navigating the perceived boundaries of their roles as students. They did not yet feel like doctors, and the experience of co-creation brought this realisation to the forefront, prompting a deeper reflection on what it truly means to be a doctor. The figured world of co-creation also created a space where their present student identity and the aspirational figured world of the medical profession existed at the same time, not necessarily comfortably:

“*I am just a student that is trying my best to pick up traits of what a good doctor is, that involves a lot of like experience, and exposing yourself to different kinds of doctors, and then picking up the good traits. But how “belong” I feel to this community of doctor still, not there yet*.” (P03, Third Interview)

The reflective journal entries revealed a growing awareness of the implicit expectations and norms tied to their current identities as students, alongside the gradual emergence of an aspirational educator identity. As they navigated this boundary, they had to negotiate their place within this figured world of co-creation in medical education and the inherent tensions it presented within the figured world of curriculum governance, where power to effect change remained concentrated among faculty and institutional actors.

One student wrote, “*My views and feedback only take into account students’ perspectives, which is just a small aspect of the larger scheme of things. There are the practical aspects of implementing changes, the reason for why changes have not been brought thus far, the reasons for the current systems and etc. Thus, I feel that my chance of influencing change concretely to the current curriculum may be rather slim*.” (P01, Reflection Journal)

Similarly, another student questioned whether her role in the co-creation process was one where she was a senior year student, a collaborator with faculty, or her status as a student participant. She was simultaneously occupying and authoring an identity within several overlapping figured worlds, each with its own social positioning and expectations. The student wrote, “*I feel like I’m torn between should I suggest this thing that I think would be helpful, but then I don’t know if it will like make my juniors’ life harder*,” (P05, Third Interview) These reflections highlighted the constant negotiation and authoring of roles that students experienced as they grappled with the limits of their influence while cautiously stepping into roles where they could contribute to meaningful change.

## Discussion

We explored how medical students negotiate their roles, boundaries, and tensions within the figured world of co-creation.

Students initially struggled to view themselves as legitimate contributors but, at the same time, participation in co-creation opened up possibilities that they had not previously imagined for themselves: the possibility of influencing curriculum, of being heard, and of beginning to see themselves as more than passive recipients of education. To do so, they had to constantly negotiate powerful contextual and institutional forces [22], including structural hierarchies, assessment cultures, and longstanding norms around expertise that shaped what felt permissible.

Students did not simply author new identities within clearly defined roles in the co-creation process. Instead, their identities shifted fluidly as they continually recalibrated their positionality and roles while navigating shifting expectations and power relations. They were at times deferential learners, and at others assertive collaborators, and occasionally something closer to a reflective future doctor. Seen through a Figured Worlds lens, these movements can be understood as responses to evolving expectations, relationships and power dynamics within the co-creation context. This dynamic resonates with the literature on proto-professionalism (e.g., [23]) and professional identity formation (e.g., [24]), which frame professional identity as developmental. We tentatively suggest that co-creation gives students a view outside of the boundaries of their current figured worlds and into future, potential figured worlds and identities that they consider building towards.

The data highlight that students’ engagement in co-creation is shaped by how safe they feel to contribute and by whether their voices are recognised as carrying genuine influence [25]. Supporting co-creation therefore involves making role expectations and boundaries explicit, so that faculty, clinical educators and students understand the possibilities and limitations of their agency [26]. We also observed that the constant shifting of positionalities and identities may carry a certain degree of emotional labour [27]. These affective dimensions appear to be a critical part of the co-creation process, suggesting that the emotional work involved in navigating roles and relationships warrants greater attention.

In terms of suggestions for future practice, mentorship, guided reflection, and open dialogue may help students navigate power differentials and support their professional and personal development through the co-creation process. The findings further suggest that legitimising students’ roles as co-creators requires not just symbolic inclusion but also clear guidelines that delineate responsibilities, and possibly decision-making mechanisms through which student contributions are valued and acted upon [7,28]. It would also be useful to study faculty views of the co-creation process.

In Singapore, power distance is high [29]. Students are socialised to defer to authority and view faculty as unquestioned experts. These assumed norms may make co-creation in medical education particularly complex, inhibiting students from speaking up or challenging the status quo [30]. Students in a different context, one with less power differentiation, may have been less surprised at having their contributions taken seriously. Similarly, student motivation to participate in co-creation as a way of giving something back to the School, to contribute to the collective good rather than for individual recognition, may reflect broader societal values in the collectivist culture of Singapore [31]. This motivator may be less apparent in more individualistic cultural contexts. Further research and comparative studies examining cultural and institutional influences would help to advance our understanding of co-creation across diverse contexts.

### Strengths and Limitations of this study

Our use of a theoretical framework for secondary data analysis aids analytical (conceptual) generalisability [32]. The specific choice of Figured Worlds theory offers valuable insights into how medical students negotiate the dynamics of co-creation, particularly in illuminating roles, boundaries and power relations within institutional hierarchies. Alternative theoretical frameworks may, of course, highlight other aspects of co-creation [33]. Although our participants are few in number, as individuals involved in co-creation processes, they had high information power [32].

As discussed earlier, the co-creation experience may differ in other contexts with lower power distance. Similarly, that the study was associated with a particular curricular context (the Scholarly Project) may have shaped participants’ responses even though we made very clear that taking part in this research was voluntary. We have no way of knowing if the students who expressed interest (via their ranking) in the co-creation Scholarly Projects were representative of the medical student body and thus we do not know if their views and experiences would be widely held. We hoped that our use of near-peer interviewers would have aided open and frank discussion.

## Conclusion

Experiencing tensions when negotiating the figured world of co-creation is not necessarily bad. Rather, co-creation could be a productive space for students to gain deeper insights into their roles as learners and provide opportunities for identity development and professional growth. The success of co-creation initiatives is likely to depend on how effectively institutions can support students in negotiating their roles, boundaries and tensions. Institutions and individuals should acknowledge both the opportunities and challenges that this negotiation presents.

## Supplementary Appendix 1

Before commencing project:

- What do you know about the principles of co-creation? How do you feel about it? What do you expect to get out of your participation in it?
- How would you describe the roles of student and faculty in medical education?
- How would you describe the student-faculty relationship in medical education?
- How do you think your medical education so far has prepared you for being a doctor? Which elements of your education do you see as being most important?
- How do you see your future role as a doctor? How do you see your place in the clinical community?

After project completion:

- How did you feel throughout the co-creation process? Any major changes along the way? What made you feel this way?
- How would you now describe the roles of student and faculty in medical education? How did being involved in co-creation affect your views?
- How would you now describe the student-faculty relationship in medical education? How did being involved in co-creation affect your views?
- How do you think your medical education prepares you for being a doctor? Which elements do you see as being most important? How did being involved in this co-creation project affect your views? How will you build on these elements in the future to prepare yourself for being a doctor?
- How do you see your future role as a doctor? How do you see your place in the clinical community? How did being involved in co-creation affect your views?
- What improvements would you suggest to the co-creation process?

After returning to clinical studies:

- Have you applied any knowledge or skills gained from co-creation in your clinical studies? If so, how did you do it?
- Have you explored any new roles that you identified during your co-creation experience? If so, could you tell me more?
- How did your medical education prepare you for being a doctor? Which elements were the most important? How did your co-creation experience affect your preparation?
- How would you describe your relationship with your clinical colleagues? How did your co-creation experience affect the way you approach these relationships?
- How do you see your future role as a doctor? How do you see your place in the clinical community? Do you feel competent in this role?
- Any other comments on how being involved in co-creation has affected your progress as a medical student?

## Supplementary Appendix 2

1. What are your motivations to do well in this module and the co-creation project?
2. How confident are you that you would perform well in this module and do a good job in the co-design project?
3. How well would you rate your grasp of the content of this module and your know-how of the co-creation process?
4. How easy do you feel that you are able to take initiative, participate in discussion, and share ideas without any apprehension?
5. Do you feel that you are being treated as an equal by the content experts?
6. Do you feel that you have received support from other students or that you have provided support to other students in this co-creation process?
7. Do you feel welcome and safe in this co-creation environment and the content experts were approachable and encouraging?
8. Do you take the initiative in the discussion, ask questions, share suggestions, and find it enjoyable doing so?
9. How would you describe your chances of influencing change in this module, such as shaping its learning goals, assessment method, or etc?
10. How well are you able to choose what content to go into the co-design module, that you feel are more aligned with your own interest, the learning goals of the module and etc?
